# Cyclic Peptide-Gadolinium Nanocomplexes as siRNA Delivery Tools

**DOI:** 10.3390/ph14111064

**Published:** 2021-10-20

**Authors:** Amir Nasrolahi Shirazi, Muhammad Imran Sajid, Dindyal Mandal, David Stickley, Stephanie Nagasawa, Joshua Long, Sandeep Lohan, Keykavous Parang, Rakesh Kumar Tiwari

**Affiliations:** 1 Department of Pharmaceutical Sciences, College of Pharmacy, Marshall B. Ketchum University, Fullerton, CA 92831, USA; davidstickley.2021@ketchum.edu (D.S.); stephanienagasawa.2021@ketchum.edu (S.N.); joshualong.2021@ketchum.edu (J.L.); 2 Center for Targeted Drug Delivery, Department of Biomedical and Pharmaceutical Sciences, Harry and Diane Rinker Health Science Campus, Chapman University School of Pharmacy, Irvine, CA 92618, USA; sajid@chapman.edu (M.I.S.); dmandal@kiitbiotech.ac.in (D.M.); lohan@chapman.edu (S.L.); parang@chapman.edu (K.P.); 3 Faculty of Pharmacy, University of Central Punjab, Lahore 54000, Pakistan; 4 School of Biotechnology, KIIT Deemed to be University, Bhubaneswar 751024, India

**Keywords:** siRNA delivery systems, cyclic peptides, gadolinium nanoparticles, intracellular transportation, nanocomplexes

## Abstract

We have recently reported that a cyclic peptide containing five tryptophan, five arginine, and one cysteine amino acids [(WR)_5_C], was able to produce peptide-capped gadolinium nanoparticles, [(WR)_5_C]-GdNPs, in the range of 240 to 260 nm upon mixing with an aqueous solution of GdCl_3_. Herein, we report [(WR)_5_C]-GdNPs as an efficient siRNA delivery system. The peptide-based gadolinium nanoparticles (50 µM) did not exhibit significant cytotoxicity (~93% cell viability at 50 µM) in human leukemia T lymphoblast cells (CCRF-CEM) and triple-negative breast cancer cells (MDA-MB-231) after 48 h. Fluorescence-activated cell sorting (FACS) analysis indicated that the cellular uptakes of Alexa-488-labeled siRNA were found to be enhanced by more than 10 folds in the presence of [(WR)_5_C]-GdNPs compared with siRNA alone in CCRF-CEM and MDA-MB-231 cells after 6 h of incubation at 37 °C. The gene silencing efficacy of the nanoparticles was determined via the western blot technique using an over-expressed gene, STAT-3 protein, in MDA-MB-231 cells. The results showed ~62% reduction of STAT-3 was observed in MDA-MB-231 with [(WR)_5_C]-GdNPs at N/P 40. The integrity of the cellular membrane of CCRF-CEM cells was found to be intact when incubated with [(WR)_5_C]-Gd nanoparticles (50 µM) for 2 h. Confocal microscopy reveals higher internalization of siRNA in MDA-MB-231 cells using [(WR)_5_C]-GdNPs at N/P 40. These results provided insight about the use of the [(WR)_5_C]-GdNPs complex as a potent intracellular siRNA transporter that could be a nontoxic choice to be used as a transfection agent for nucleic-acid-based therapeutics.

## 1. Introduction

Signaling pathways are a chain of reactions that precisely regulate gene expression and lead to a controlled cell function. Within this process, RNA interference (RNAi) modulates the gene expression process through degradation [1]. In a cellular medium, a small double-strand RNA molecule (siRNA) moderates RNAi through association with the RNA-induced slicing complex (RISC). However, as a therapeutic entity, siRNA is a class of RNA molecules that elicits the knockdown of its complementary target mRNA and consequently inhibits the expression of the corresponding protein [2]. The nature of siRNA is therapeutically unique and offers multiple advantages, such as specificity, lower toxicity, and high availability [3,4]. However, siRNA suffers from several drawbacks, including a high level of instability in the presence of peptidases, a high incidence of nonspecific interactions with proteins, and low cellular internalization capability. Most siRNA delivery systems have been used to facilitate siRNA’s internalization process and get through the cell membrane via energy-dependent endocytosis [5,6,7].

A wide range of nonviral delivery systems including chitosan [8,9,10,11], dendrimers [12,13], polyethyleneimines [14,15], gold nanoparticles [16,17], iron oxide nanoparticles [18], silica-based nanoparticles [19], quantum dots [20], cell-penetrating peptides [21], and nanogels [22] have been used for transporting siRNA molecules. One of the major methods to enhance siRNA delivery efficiency is to employ hydrophobic particles to encapsulate them. The United States Food and Drug Administration (FDA) approved a lipid-based nanoparticle system for encapsulating siRNA in the treatment of polyneuropathy in patients with hereditary transthyretin-mediated amyloidosis [23]. Therefore, there is a significant potential to enhance the delivery efficacy of siRNA therapeutics into cells.

To this date, gadolinium-based particles (GdNPs) showed promising potential. The FDA have approved them for MRI imaging in cancer patients [24]. Naturally, due to the specific chemistry of gadolinium, it can promote MRI images by reducing *T1* relaxation constant when it gets accumulated into tissues [25]. Furthermore, *T1* contrast agents such as Gd-DTPA (Magnevist) and Gd-DOTA (Dotarem) are great examples of where gadolinium is able to create stable complexes via chelation with different ligands. One of the major drawbacks of gadolinium complexes is the leaching issues of the nonmetabolizable Gd^3+^ ions. The leaked gadolinium could be accumulated in an organ such as kidney, causing severe toxicity, leading to renal toxicity [26]. Although gadolinium particles have been widely used in imaging procedures, their liposome-based formulations have been used for diagnosis and chemotherapy outcomes [27,28]. Liposomes with dual functionality of encapsulating doxorubicin and Gd through drug–metal complexation offered a higher accumulation of Gd particles and doxorubicin in target tissues. Additionally, gadolinium-doped layered double hydroxide/Au nanocomposites exhibited high doxorubicin loading capacity with a pH-responsive release profile, leading to a higher release profile in the cytoplasm [29]. Thus, the development of stable gadolinium nanoparticles (GdNPs) is a major area of research interest.

Peptide-based delivery systems are one of the widely used platforms that were utilized to assist non-permeable drugs and biologically important molecules in crossing cell membranes [30]. Our interest has been involved in developing novel peptide-based carriers to deliver siRNA intracellularly [31,32].

Cell-penetrating peptides (CPPs) were discovered to transport siRNA into cells efficiently. Among them, cyclic peptides were found to offer multiple advantages compared to their linear counterparts, including higher enzymatic stability and higher penetrating properties due to their optimized orientation of engaged amino acids [33,34,35]. Cyclic peptide-capped gold nanoparticles showed higher efficiency in delivering a model siRNA compared to that of lipofectamine [36]. We also found that the intracellular transporting potency of cyclic peptides could be improved when combined with metal nanoparticles [37,38].

The specific orientation of engaged, positively charged, and hydrophobic amino acids in the structure of the peptide create an optimized complex to interact and internalize into the cell membrane. The combination of hydrophobic forces, positive charge, and nanoparticles can be used for the delivery of siRNA since this molecule contains negatively charged groups. As elaborated, metal nanoparticles and CPPs were found to be efficient tools for the delivery of siRNA. Thus, we decided to take advantage of a system containing both GdNPs and CPP to obtain an optimized outcome. Here, we evaluated a complex containing a cyclic CPP with arginine, tryptophan, and cysteine with biofriendly GdNPs for the delivery of siRNA. The potency of CP-GdNPs was examined for the delivery of siRNA in cell-based assays.

## 2. Results and Discussions

### 2.1. Preparation and Characterization of Peptide-GdNPs

**Figure 1:**
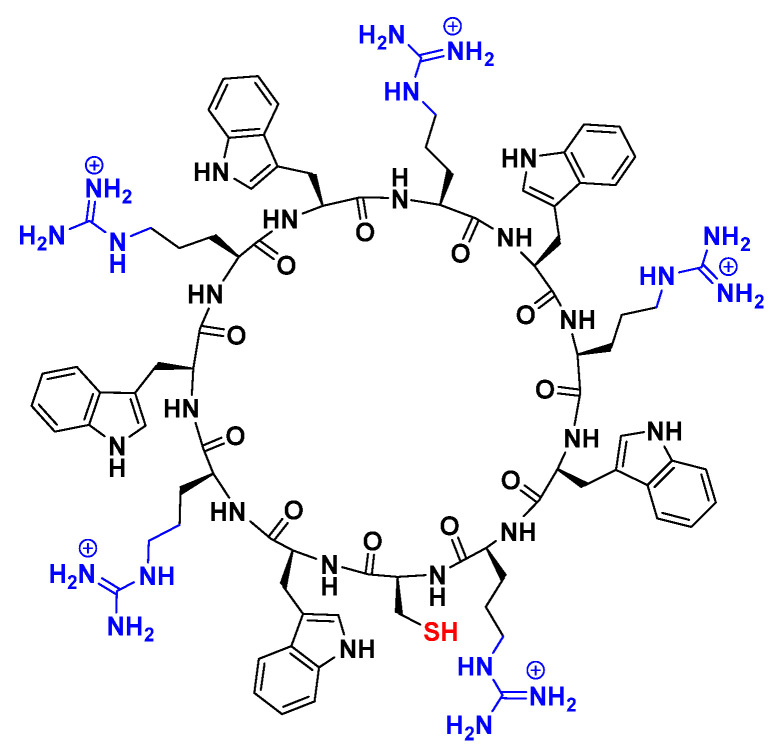
Chemical structures of the cyclic peptide [(WR)_5_C] **1**.

A cyclic peptide containing eleven amino acids [CWRWRWRWRWR] [(WR)_5_C] (**1**) was synthesized using solid-phase peptide chemistry (Figure 1) according to the previously reported procedure [39].

**Figure 2:**
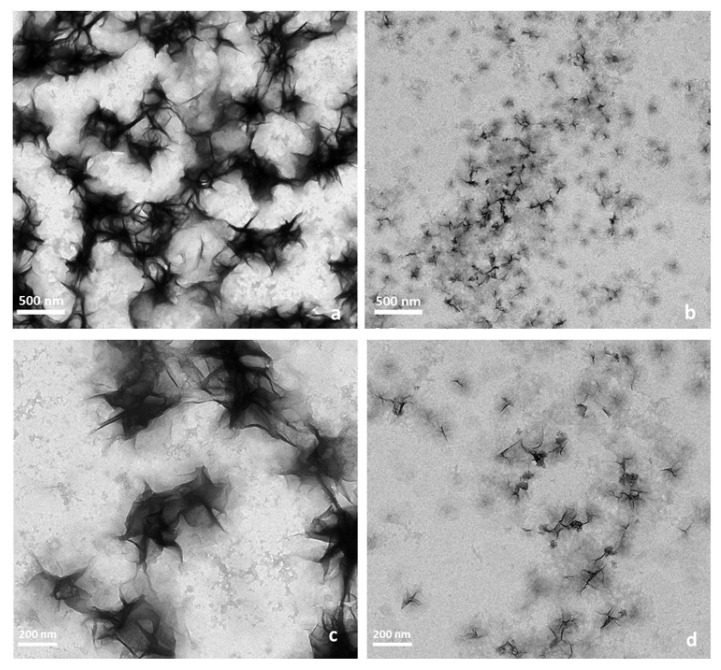
TEM images of [(WR)_5_C]-GdNPs (**a**,**c**), [(WR)_5_C]-GdNPs-loaded siRNA(**b**,**d**).

The structure and morphology of [(WR)_5_C]-GdNPs were investigated using transmission electron microscopy (TEM) as recently reported by us [39]. It was found that [(WR)_5_C]-GdNPs are presented as star-shape particles with coacervates structure in size range of 240–260 nm. It was proposed that the involved amino acids in the structure generated and capped GdNPs. This behavior of the peptide was due to the presence of positively charged arginine and hydrophobic tryptophan amino acids. Figure 2a,c demonstrates the morphologies of [(WR)_5_C]-GdNPs as previously reported at a lower magnification [39]. However, it was observed that upon mixing [(WR)_5_C]-GdNPs with the siRNA molecule, Figure 2b,d, the structure was changed. As it is shown, the space between gadolinium nanoparticle becomes wider and generate cavities suggesting the incorporation of siRNA. At the same time, the metal nanoparticles become smaller after the engagement with the siRNA molecules. Alternatively, the star-shaped nanoparticle formation could be due to aggregated sheets formation. Additional investigations are required to study the behavior of the nanoparticles combined with siRNA to discover the involved forces.

### 2.2. Formation of Peptide-–Nanoparticles and siRNA Complexes and Their Binding Affinity

**Figure 3:**
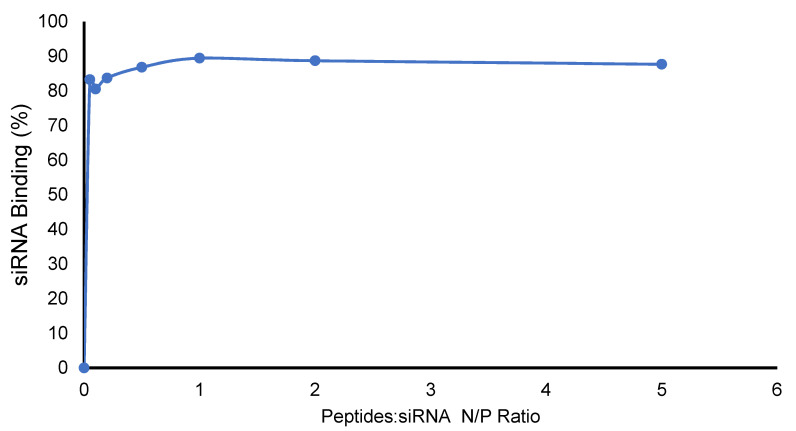
Binding affinity of the peptide to scrambled siRNA, indicating the percentage of siRNA bound to the peptide at different N/P ratios.

Peptide–nanoparticles were mixed with siRNA to make peptide–nanoparticle–siRNA complexes. The concentration of siRNA was kept fixed in all the experiments, while the peptide concentration was increased to increase the N/P ratio. The binding capacity of the scrambled siRNA with the peptide was determined by the SYBR Green II dye exclusion method. Finally, the ratio required for 50% binding (BC50) was calculated based on the line equation of the linear portion of the curve under these experimental conditions. The BC50 was determined to be 0.044, showing the existence of a decent binding affinity between the siRNA and peptide-based GdNPs from a set of triplicates in the experiment (Figure 3). Multiple elements, including positively charged arginine amino acids in the structure of the peptide and the negatively charged phosphates of the siRNA could be involved in the binding affinity. The results obtained are consistent with the previous reports of tryptophan- and arginine-containing peptides from our group [40,41].

### 2.3. Zeta Potential of Peptide-GdNPs

**Figure 4:**
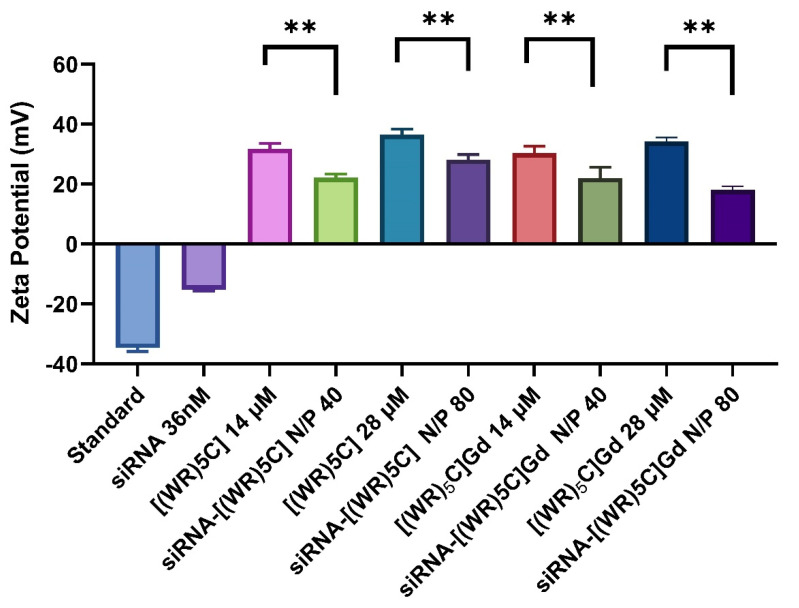
Zeta potential of [(WR)_5_C] and [(WR)_5_C]-GdNPs at different concentrations alone and complexed with siRNA at different N/P ratios (if *p* < 0.01 then **).

The Zeta potential values of the [(WR)_5_C] and [(WR)_5_C]-GdNPs alone was determined according to the concentrations that were used in making complexes with siRNA (Figure 4). The results indicated that [(WR)_5_C] at 14 µM (corresponding to the concentration of the peptide in the complex at N/P 40) has a zeta potential value of +31.7 mV and the zeta potential for [(WR)_5_C] at 28 µM (corresponding to the concentration of the peptide in the complex at N/P 80) was 36.5 mV. The zeta potential values of [(WR)_5_C]-GdNPs at 14 µM and 28 µM were 30 mV and 34 mV, respectively. Compared to the [(WR)_5_C] peptide, the zeta potential values of [(WR)_5_C]-GdNPs were a slightly reduced (Figure 4). This slight decrease could be attributed to the structure and assembly of [(WR)_5_C]-Gd. A similar trend was observed in the complexes of the peptides with siRNA. For instance, the siRNA alone showed a ZP of approximately −15 mV; the negative charge of the ZP is due to the presence of phosphate groups in the siRNA. Both [(WR)_5_C] and [(WR)_5_C]-Gd showed a positive zeta potential value that can be attributed to the presence of positively charged amino groups of arginine in the peptide structure. Furthermore, the Zeta potential value points to the stability of the complexes in the solution form.

### 2.4. Cytotoxicity of Peptide-GdNPs

**Figure 5:**
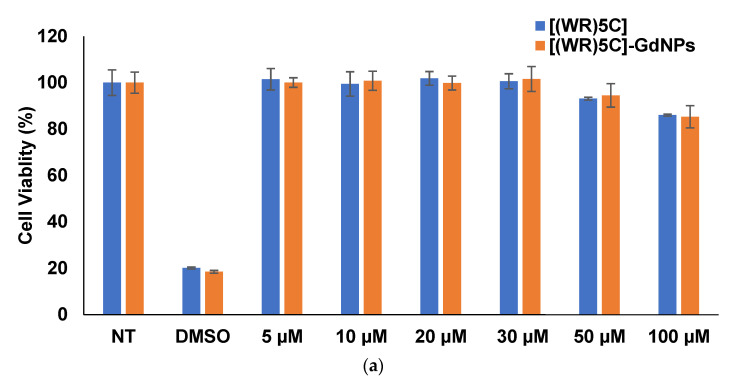

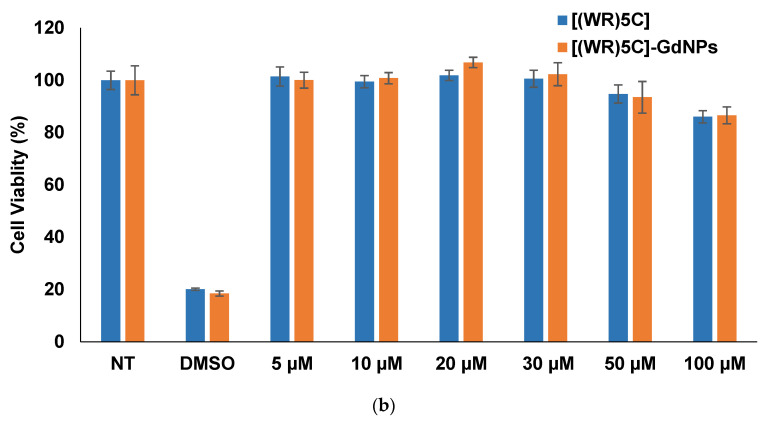
Cytotoxicity of the cyclic peptide [(WR)_5_C] and corresponding [(WR)_5_C]-GdNPs in (**a**) CCRF-CEM cells and (**b**) in MDA-MB-231 cells after 48 h of incubations.

The toxicity of [(WR)_5_C]-GdNPs was evaluated in human leukemia (CCRF-CEM) and triple-negative breast cancer (MDA-MB-231) cells. The cytotoxicity assay was performed by standard MTS method in which the cells were treated with varying concentrations in a range of 5 to 100 µM and the cells were incubated for 48 h. The results indicated that the peptide and peptide-based gadolinium nanoparticles, [(WR)_5_C]-GdNPs did not exhibit significant cytotoxicity (~93% cell viability at 50 µM) in CCRF-CEM and MDA-MB-231 cell lines (Figure 5a,b).

### 2.5. Evaluation of Peptide-GdNPs as Molecular Transporters

**Figure 6:**
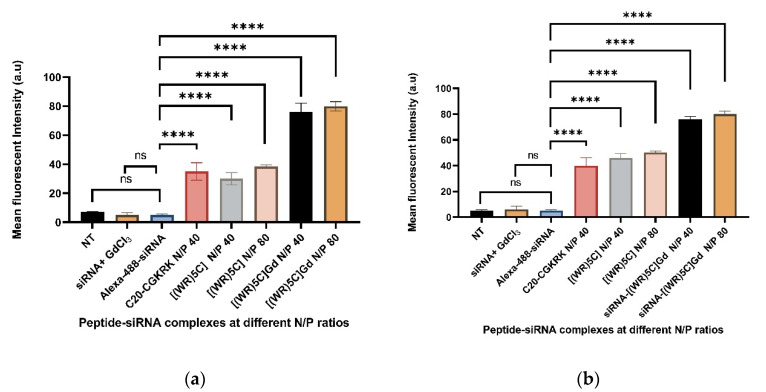
(**a**) Cellular uptake of Alexa-488 siRNA in the presence of [(WR)_5_C] and [(WR)_5_C]-GdNPs at different ratios after 6 h incubation in (**a**) CCRF-CEM cells (ns = non-significant, if *p* < 0.0001 then ****) and (**b**) in MDA-MB-231 cells (ns = nonsignificant, if *p* < 0.0001 then ****).

To monitor the ability of peptide-GdNPs for the delivery of siRNA molecules, a model experiment using Alexa-488-labeled scrambled siRNA was designed. Using the flow cytometry method, the intracellular uptake of Alexa-488-labeled scrambled siRNA was quantified in the absence and presence of [(WR)_5_C]-GdNPs. Traditionally, the intracellular delivery of siRNA has been challenging due to the specific size of nucleic-acid-based drugs and the presence of negatively charged groups. Here, the delivery of a model siRNA (36 nM) was evaluated when combined with [(WR)_5_C]-GdNPs and compared with that of siRNA alone in CCRF-CEM and MDA-MB-231 cells and different N to P ratios, where N represents the number of moles of ionizable nitrogen in the peptide and P represents the number of moles of phosphate in the siRNA. Gadolinium particles alone, free Alexa-labeled siRNA, and the nontreated cells were used as a negative control, while previously reported C20-CGKRK [25] peptide was used as a positive control for this assay. The analysis of fluorescence-activated cell sorting (FACS) results showed that the cellular uptake of Alexa-488 siRNA was enhanced when loaded on [(WR)_5_C]-GdNPs by 10 folds compared to siRNA alone in CCRF-CEM and MDA-MB 231 cells (Figure 6a,b). It should be mentioned that [(WR)_5_C]-GdNPs and the siRNA were mixed and incubated for 30 min to increase their binding affinity. FACS data suggested that [(WR)_5_C]-GdNPs could potentially be used as a carrier system to deliver siRNA intracellularly. Moreover, gadolinium particles alone did not show any potential to deliver siRNA into cells (both CCRF-CEM and MDA-MB-231 cells), suggesting that the presence of the cyclic peptide is vital for their function.

As it is shown in Figure 6a, the cellular uptake of the siRNA did not improve in the presence of gadolinium chloride alone, significantly suggesting that the presence of peptide is necessary for their function. At the same time, peptide alone improved the uptake of siRNA compared to that of the free siRNA. However, the [(WR)_5_C]-GdNPs complex enhanced the siRNA uptake by a factor of two compared to the peptide alone. This part of the results suggested that the presence of both the peptide and gadolinium nanoparticles are critical for the function of the system.

A similar pattern was observed in MDA-MB-231 cells. The intracellular uptake of siRNA was enhanced to the highest when peptide and gadolinium nanoparticles were combined. The results proved that although the [(WR)_5_C] peptide is necessary for intracellular uptake of the siRNA, gadolinium nanoparticles are the major elements that enhance the siRNA uptake. The availability and accessibility of multiple binding sites allow the formation of strong bonds between gadolinium nanoparticles and cyclic peptides. Combined forces induced by amino acids provide the cyclic-peptide-based nanocarrier to be able to entrap siRNA molecules efficiently. In addition, their cyclic nature equipped the carrier with a higher level of stability compared to their linear counterparts. [(WR)_5_C] contains tryptophan, arginine, and cysteine that could offer hydrophobic forces, electrostatic interactions, and covalent bindings, respectively. The presence of positively charged amino acids helps the delivery system to bind with negatively charged siRNA, and at the same time, bind with negatively charged elements in the structure of the cell membrane. Here, the presence of the cyclic peptides enhanced the intracellular uptake of siRNA compared to that of siRNA alone. This uptake was improved further when gadolinium nanoparticles were added to the complex. Moreover, it was found that the presence of the peptide alone can improve the uptake of the siRNA compared to that of siRNA alone. However, when the peptide was combined with GdNPs, the transporting efficiency was maximized, suggesting the importance of gadolinium nanoparticles. Furthermore, as it is shown in Figure 6a,b, the cellular uptake of siRNA was improved slightly by [(WR)_5_C]-GdNPs at N/P 80 compared to that of [(WR)_5_C]-GdNPs N/P 40. At the same time, the peptide alone complexes such as [(WR)_5_C] N/P 40 and [(WR)_5_C] N/P 80 were able to enhance the siRNA uptake compared to that of siRNA alone. Overall, [(WR)_5_C]-GdNPs at N/P 80 was able to improve the cellular uptake by approximately 1.5–2 folds as compared to [(WR)_5_C] at N/P 80 in both cell lines. Their transporting ability were found to be in a similar range of a fatty acylated CGKRK peptide (C20-CGKRK at N/P 40). This piece of data suggested that the cyclic peptide and gadolinium nanoparticles complexes are able to entrap and transport the siRNA molecule intracellularly. It should be mentioned that higher cellular uptake does not directly represent the bioavailability of the cargo molecule. It was previously reported that the various pathways are responsible for the cellular internalization of peptides containing arginine and tryptophan amino acids [33,34,35,36,37,38,39].

### 2.6. Protein Quantification (Western Blot)

**Figure 7:**
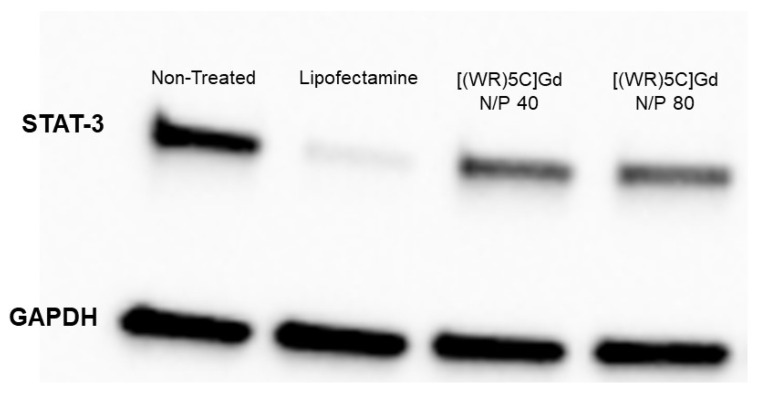
STAT-3 expression in the MDA-MB-231 cells treated with siRNA loaded with [(WR)_5_C]-GdNPs at N/P 40 and N/P 80 using western blot. GAPDH serves as a negative control, NT reflects nontreated cells, and lipofectamine serves as a positive control. Lipofectamine is one of the widely used transfection agents that was used in comparative assays with [(WR)_5_C]-GdNPs.

**Figure 8:**
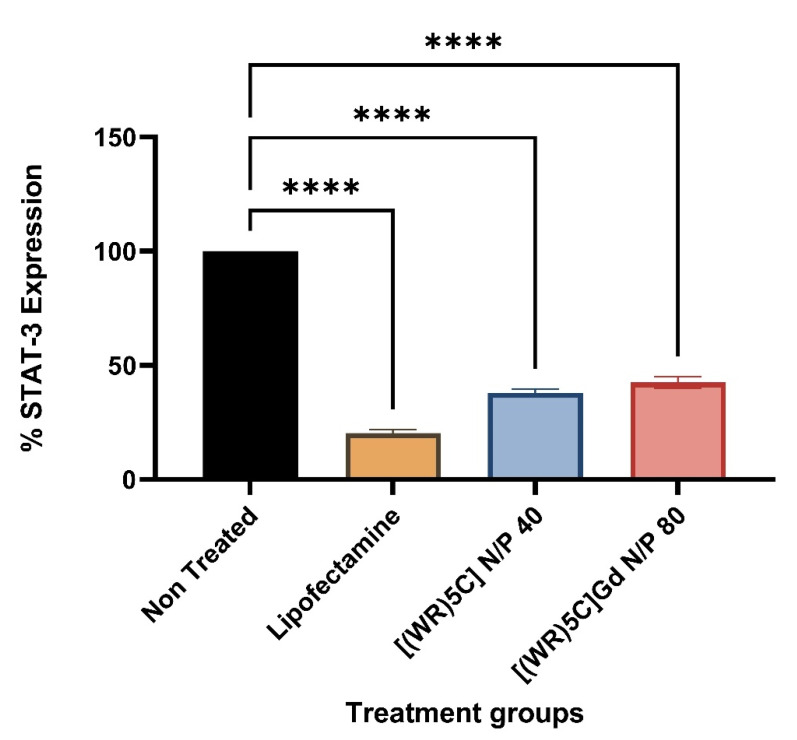
Quantification of % STAT-3 expression from the gel electrophoresis experiment in MDA-MB-231 cells.; (*n* = 3), bars represent the standard deviation (if *p* < 0.0001 then ****).

To evaluate the protein silencing efficiency of the delivered siRNA, STAT-3 protein was targeted in triple-negative breast cancer cells, MDA-MB-231. Protein silencing efficiency of control peptide and peptide-GdNPs was assessed by western blot analysis. The results indicated that [(WR)_5_C]-Gd NPs reduced the expression of STAT-3 by more than 60% when the GdNPs:[(WR)_5_C] ratio was 40. A similar pattern was observed by 55% knockdown in the presence of a [(WR)_5_C]-Gd NPs at N/P ratio of 80 (Figure 7). The results are consistent with cellular uptake findings described earlier (Figure 8).

### 2.7. Membrane Integrity

**Figure 9:**
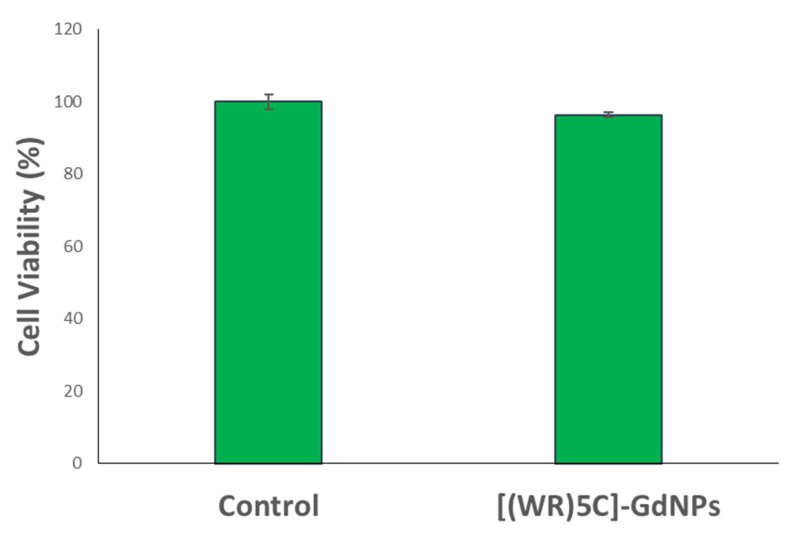
The membrane integrity of [(WR)_5_C]-GdNPs (50 µM) in CCRF-CEM cells after 2 h.

The integrity of the cellular membrane of CCRF-CEM cells was evaluated after incubating with a 50 µM of [(WR)_5_C]-GdNPs for 2 h. The presence of rupture or any other morphological changes in the structure of the membrane was monitored and counted using trypan blue. The number of intact cells and their morphology did not reveal any significant difference with those of control cells (Figure 9). The results of this assay showed that although [(WR)_5_C]-GdNPs function as an efficient transporter, they did not damage the membrane integrity.

### 2.8. Confocal Microscopy Imaging

**Figure 10:**
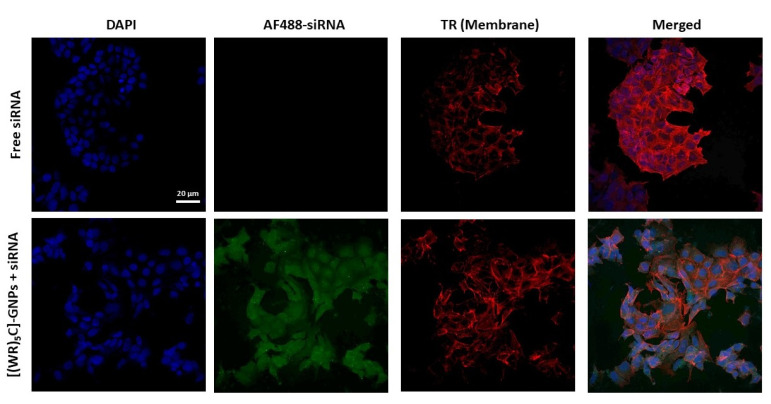
Confocal microscope images of Alexa 488-siRNA uptake by MDA-MB-231 cells in the presence of [(WR)_5_C]-GdNPs after 2 h incubation. AF488 represents Alexa fluor 488 and TR represents Texas Red.

Further confocal microscopy imaging was conducted to visualize the intracellular uptake of Alexa 488-siRNA. MDA-MB-231 cells were treated with Alexa 488-siRNA alone and Alexa 488-siRNA/[(WR)_5_C]-GdNPs complex at N/P 40. The results were compared and visualized using confocal microscopy (Figure 10). Cells that were treated with Alexa 488-siRNA/[(WR)_5_C]-GdNPs showed a higher fluorescence signal compared to that of Alexa 488 alone, suggesting that peptide-based gadolinium nanoparticles facilitate the internalization process. A Texas Red phalloidin solution and DAPI were used to stain the cell membrane and nucleus, respectively. As shown in Figure 10, the siRNA alone was not able to internalize to the selected cells. However, the presence of [(WR)_5_C]-GdNPs enhanced the siRNA uptake in MDA-MB-231 cells.

## 3. Materials and Methods

Starting material for the preparation of peptide-based gadolinium nanoparticles was previously described [39]. Silencer^®^ Negative Control #1 siRNA, Catalog #: AM4635-AM4636 was purchased from Applied Biosystems, Ambian Inc., USA. Negative control siRNAs, the siRNAs with sequences that do not target any gene product—are essential for determining siRNA transfection efficiency and to control for the effects of siRNA delivery. We used this siRNA for performing binding affinity, DLS experiments, and flow cytometry experiments. In addition, Hs_STAT3_7 FlexiTube siRNA was purchased from QIAGEN LLC, USA. The target sequence was 5′-CAGCCTCTCTGCAGAATTCAA-3′, the sense strand was 5′-GCUUCUCUGCAGAAUUCAATT-3′ and the antisense strand was 5′-UUGAAUUCUGCAGAGAGGCTG-3′. STAT-3 (124H6) Mouse mAb #9139 and GAPDH (D4C6R) Mouse mAb #97166 were purchased from Cell Signaling Technology (CST), USA for the western blotting experiment. Horseradish peroxidase (HRP)-linked secondary antibody was purchased from Abcam Inc., USA. Mini-PROTEAN TGX stain-free precast gels and Trans-Blot Turbo Mini 0.2 µm PVDF transfer packs #1704156 were purchased from Bio-Rad Inc., Hercules, CA, USA. All other reagents used for western blotting experiments were purchased from Bio-Rad Inc., Hercules, CA, USA.

### 3.1. Synthesis of Cyclic Peptide [CWRWRWRWRWR] (**1**)

The peptide was synthesized according to the previously reported procedure using Fmoc/tBu solid-phase peptide synthesis. The peptide was purified by reverse-phase high-performance liquid chromatography (RP-HPLC) and confirmed by matrix-assisted laser desorption/ionization time of flight (MALDI-TOF) mass spectrometry [39].

### 3.2. In Situ Preparation of Cyclic [(WR)_5_C]-GdNPs

The in situ synthesis of peptide-capped GdNPs was conducted in the aqueous solution. Two solutions, including 1 mL of [(WR)_5_C] (10 mM) and 1 mL of GdCl_3_ (10 mM) in water, were mixed within a glass container. The mixture was mixed for 2 min using a vortex. The color and temperature of the product did not change upon mixing. The mixed solution was monitored for 8 h and remained colorless.

### 3.3. Complex Formation of Cyclic [(WR)_5_C]-GdNPs/siRNA Complexes, Binding Affinity, and BC50

The complex of [(WR)_5_C]-GdNPs and siRNA were formed through physical mixing. Appropriate volumes of [(WR)_5_C]-GdNPs and siRNA solution was mixed in presence of HBSS buffer to obtain the different N/P ratios. siRNA concentration was constant at 36 nM whereas the maximum concentration of [(WR)_5_C]-GdNPs was 28 µM. The concentration of [(WR)_5_C]-GdNPs is well below the cytotoxic concentration. N to P ratio is commonly used to determine the ratio of the peptide to siRNA. N refers to the number of moles of ionizable nitrogen present in the peptide whereas the P represents the number of moles of phosphate present in the siRNA [32]. The ratio was calculated using the formula below:

$$\frac{N}{P}=\frac{\left(number\;of\;moles\;of\;peptide\times number\;of\;ionizabale\;nitrogens\right)}{\left(number\;of\;moles\;of\;siRNA\times 48\right)}$$

The mixture was prepared through vigorous physical mixing via vortex. The mixture complex was incubated for 30 min before any assays to obtain a satisfactory binding.

The binding capacity of the scrambled siRNA (AM4635 negative control siRNA No 1, Thermo Fisher Scientific, Waltham, MA, USA) with the peptide was determined by SYBR Green II dye exclusion method [32]. Briefly, the stock solutions of 5 µM and 50 µM of the peptides was prepared using RNase free water. Also, the solution of scrambled siRNA (892.5 nmol/L) was prepared using DNase/RNase free water. siRNA peptide complexes were prepared at N/P ratios 0, 0.05, 0.1, 0.2, 0.5, 1, 2, 5, 10, 20, and 40 in 1 mL tubes according to the previously reported procedure [42].

After a 30 min incubation at room temperature to make complexes of siRNA with peptide nanoparticles, 200 μL of SYBR Green II solution (1:10,000 dilution in water) was added to the complexes. The fluorescence of the samples was quantified in a 96-well black plate (λ excitation: 485 nm, λ emission: 527 nm) to determine the proportion of unbound siRNA. The percentage of siRNA bound to the peptide was plotted against the peptide/siRNA (N/P) ratio. Finally, the ratio required for 50% binding (BC50) was calculated based on the line equation of the linear portion of the curve under these experimental conditions. To calculate the percentage of siRNA bound to peptide-based gadolinium nanoparticles, the following equation was used

$$\%siRNA\;binding=100-\frac{fluorescence\;signal\;of\;the\;complex}{fluorescence\;signal\;for\;free\;siRNA}\times 100$$

### 3.4. Zeta Potential

Zeta potential was measured for [(WR)_5_C], [(WR)_5_C]-GdNPs, and its complexes with siRNA at 40 V using disposable folded capillary cells (DTS1070 Malvern Nano ZS Zetasizer, Westborough, MA, USA). The calibration of the instrument was performed by transfer standard DTS 1235. The zeta potential of siRNA alone at 36 nM concentration was determined. The same concentration of siRNA was used in all the subsequent analyses. N/P ratio 40 and 80 were selected for the determination of the zeta potential of siRNA complexes with the [(WR)_5_C] and [(WR)_5_C]-GdNPs. The concentration of the peptides was 14 µM in the case of N/P 40, while it was 28 µM in the case of N/P 80. Individual zeta potentials were determined through the Smoluchowski approximation. All measurements were performed at least three times, and results were checked against the quality standard of the instrument.

### 3.5. Transmission Electron Microscopy (TEM)

TEM imaging of [(WR)_5_C]-GdNPs samples were done according to our reported method [39]. The [(WR)_5_C]-GdNPs samples were obtained by using a drop of a solution of [(WR)_5_C]-GdNPs (5 mM) in water. The drop was placed on an ultrathin carbon type-A 400-mesh copper grid and left overnight to dry. The process of imaging was carried out by EAG Laboratories (Sunnyvale, CA, USA) using a FEI Techni TF-20 operated at 200 kV in bright-field TEM mode.

### 3.6. Cell Culture

CCRF-CEM cells (ATCC No. CCL-119) and MDA-MB-231 cells (ATCC No. HTB-26) were purchased from American Type Culture Collection (ATCC) (Manassas, VA, USA). Cell culture supplies were provided by Fisher Scientific (Pittsburgh, PA, USA). RPMI-16 and DMEM media were used for the proliferation of CCRF-CEM and MDA-MB-231 cells, respectively. The medium was supplemented with fetal bovine serum (FBS, 10%) and penicillin–streptomycin solution (10,000 units of penicillin and 10 mg of streptomycin in 0.9% NaCl, 1%). The cells were incubated in a humidified atmosphere of 5% CO_2_ and 95% air at 37 °C.

### 3.7. Cytotoxicity Assay of [(WR)_5_C]-GdNPs

CCRF-CEM (50,000) and MDA-MB-231 (10,000) cells were seeded separately in a 96-well plate in 100 µL of media. They were incubated for 24 h before the experiment. Since the CCRF-CEM cells are nonadherent, the RPMI medium was not replaced. After the cells had settled down, the compounds including [(WR)_5_C], [(WR)_5_C]-GdNPs, and GdNPs were added to the cells at various concentrations (5 µM to 100 µM). A similar procedure was carried out in MDA-MB-231 cells. After treatment, the plates were incubated for 48 h at 37 °C in a humidified atmosphere of 5% CO_2_. Following 48 h of treatment, 20 µL of CellTiter 96^®^ AQueous One Solution Cell Proliferation Assay (MTS) was added to each well using a multichannel pipette. The plates were incubated for 2 h at 37 °C in a humidified atmosphere of 5% CO_2_ and 95% air. The treated plates were read at the absorbance of 490 nm using a SpectraMax M5 Spectrophotometer. The absorbance of treated cells was compared to untreated cells (as 100% viability), and the percent of cell viability was calculated for each concentration of compounds. DMSO (35%) was used as a positive control for this assay [39].

### 3.8. Flow Cytometry Studies

The flow cytometry assay was used to quantify the intracellular uptake of the Alexa-488-labeled siRNA in CCRF-CEM and MDA-MB-231 cells. A quantity of 1 × 10^7^ cells per well were seeded in a 6-well plate. The medium was selected to be RPMI in CCRF-CEM cells while it was DMEM in MDA-MB-231 cells. A solution of [(WR)_5_C]-GdNPs and a solution of Alexa-488-labeled scrambled siRNA were mixed to obtain complexes with GdNPs: [(WR)_5_C] ratios of 40 and 80. The final siRNA concentration was 36 nM, and the maximum concentration of the compounds ([(WR)_5_C] and [(WR)_5_C]-GdNPs) was 28 µM. The mixture was incubated for 30 min in an ice bath to cause the formation of a complex. Then, the complex was treated with the cells. Previously reported peptide C20-CGKRK complexed with siRNA at N/P 40 was used as a positive control, whereas nontreated cells, Alexa-488-labeled scrambled siRNA (36 nM) and GdCl_3_ -siRNA complexes, were used as negative controls. The cells were incubated with their treatments for six hours at 37 °C. The incubated cells were removed after six hours and were digested with 0.25% trypsin/EDTA (0.53 mM) for 5 min. This wash removes any membrane-bound-labeled siRNA and artificial surface binding. This process was followed by another two rounds of washing cells with PBS. Flow cytometry (BD-FACSVerse; BD Biosciences; San Jose, CA, USA) was used to run the samples. The analysis of uptake was processed based on formerly reported techniques and settings [35].

### 3.9. Protein Quantification (Western Blot)

The western blot method was used to determine the expression of the target protein. A radioimmuno precipitation assay (RIPA) buffer was used for the cell lysis process based on the standard protocol. The cells were incubated with siRNA. Trypsin was used to collect the cells after 48 h. They were centrifuged at 600–800 rpm for 5 min. The cells were filtered and washed with PBS (×3). A RIPA buffer (100 µL) was used for cells resuspension (25 µL). The lysates were incubated in ice (1h). The tubes were then centrifuged for 15 min at 12,000× *g* (at 4 °C). A BSA assay was used to determine the protein concentration. The reagent (50:1, A:B, 200 µL) was added to 25 µL of the standard sample. All samples were tested in triplicate. The rest of the assay was carried out based on our previously reported protocol [30].

### 3.10. Membrane Integrity Test

CCRF-CEM cells were plated at 1 × 10^6^ cells/well in 6-well plates. They were treated with a solution of [(WR)_5_C]-GdNPs (50 μM) in serum-free media for 2 h at 37 °C. Cells were washed twice with trypsin and PBS, after 2 h of incubation. The analysis procedure was carried out according to our formerly reported method [37].

### 3.11. Confocal Microscopy Imaging

MDA-MB-231 cells were cultured and prepared in DMEM containing 10% FBS and 1% Penicillin/Streptomycin antibiotics. In a 6-well plate, cover slips were placed, and the cells were incubated on top of that for 24 h to reach confluency. Then, the cell medium was replaced by the treatments containing AF488-siRNA alone and [(WR)_5_C]-GdNPs/AF488-siRNA complex at N/P ratio of 40. After incubating for 6 h, the cells were washed with PBS three times. A fixing solution containing 3.7% formaldehyde in PBS was added to the cells and maintained for 10 min. The cells were rinsed three times in PBS for 5 min. To stain the cell membrane, a Texas Red phalloidin solution (40 μL and 10 mg BSA in 10 mL of PBS) was added to the cells and incubated at room temperature for 1 h. The cells were then washed 3 times with PBS for 5 min. One drop of DAPI was added to a slide to stain the nucleus, and the coverslips were placed face down without air bubbles and were stored overnight, away from light to dry. Once dry, a Nikon A1R high-definition resonant scanning confocal microscope and NIS-Elements software (AR 4.30.02, 64 bit) were used to image the cells.

### 3.12. Statistical Analysis

To determine the statistical significance of the cellular uptake results and western blotting data, one way ANOVA test was carried out with Dunnett’s test for multiple comparisons using GraphPad prism version 9.1.2. For the Statistical analysis of Zeta Potential results, student t-test was employed using the same software. siRNA-treated cells were considered as controls and compared with siRNA/vehicle combinations. *p* value was determined and included in the figures.

## 4. Conclusions

A new nanosized complex containing a cyclic peptide and gadolinium nanoparticles were synthesized and fabricated for intracellular siRNA delivery. The peptide and the NPs showed an avid binding capacity to siRNA with a BC50 value of 0.044 suggesting decent binding capability. The cytotoxicity profile of the compounds evaluated to significantly non-toxic (~7% cytotoxicity) at 50 µM concentrations in both MDA-MB-231 and CCRF-CEM cells. A positive zeta potential of approximately 42 mV reflects the stability of NPs. TEM microscopy revealed the size of NPs to be in a range of 240 to 260 nm.

Furthermore, flow cytometry results showed that [(WR)_5_C]-GdNPs are efficient to transport a negatively charged cell-impermeable siRNA molecule intracellularly. [(WR)_5_C]-GdNPs showed more siRNA intracellular uptake in both MDA-MB-231 and CCRF-CEM cells compared to that of siRNA alone. The expression of STAT-3 protein was decreased in the presence of [(WR)_5_C]-GdNPs in MDA-MB-231 cells. The data indicated a significantly reduced expression of STAT-3 (>55%), which reflects the adequate intracellular uptake of intact STAT-3 siRNA with consequent degradation of STAT-3 mRNA causing the reduced expression of STAT-3 protein. This investigation offered that the cell-penetrating peptide-based gadolinium nanoparticles can be safely used as a transfection agent for siRNA and can be investigated for other nucleic-acid-based therapies such as microRNAs or CRISPR/Cas9. Further studies are needed to assess their potential as a nontoxic nucleic acid delivery agent.

## Data Availability

Data is contained within the article.
